# High-resolution diffusion pattern of human infections by *Salmonella enterica* serovar Napoli in Northern Italy explained through phylogeography

**DOI:** 10.1371/journal.pone.0202573

**Published:** 2018-08-22

**Authors:** Maria Gori, Erika Ebranati, Erika Scaltriti, Pol Huedo, Giulia Ciceri, Elisabetta Tanzi, Mirella Pontello, Gianguglielmo Zehender, Stefano Pongolini, Luca Bolzoni

**Affiliations:** 1 Department of Health Sciences, University of Milan, Milano, Italy; 2 Department of Biomedical and Clinical Sciences "L. Sacco", University of Milan, Milano, Italy; 3 Risk Analysis and Genomic Epidemiology Unit, Istituto Zooprofilattico Sperimentale della Lombardia e dell’Emilia-Romagna, Parma, Italy; 4 Department of Biomedical Sciences for Health, University of Milan, Milano, Italy; 5 CRC-Coordinated Research Center “EpiSoMI”, University of Milan, Milano, Italy; Laboratoire National de Santé, LUXEMBOURG

## Abstract

*Salmonella enterica* serovar Napoli (serovar Napoli) is an emerging cause of human salmonellosis in Northern Italy. No specific reservoirs of serovar Napoli have been identified in Italy, so far. However, the environment, especially surface waters, has been hypothesized as an important source of infection based on the observation that genotypically different clusters of serovar Napoli are detected in different geographical macro-areas. To further support the hypothesis of a spatially-restricted pattern of serovar Napoli diffusion, a spatial segregation of serovar Napoli lineages should be observed also at smaller geographical scale. However, classical genotyping techniques used for *Salmonella*, such as pulsed-field gel electrophoresis (PFGE), did not possess enough discriminatory power to highlight spatial clustering of serovar Napoli within the macro-areas. To this purpose, we performed phylogeographical analyses based on genome-wide single nucleotide polymorphisms to test whether spatio-temporal evolution patterns of serovar Napoli in Northern Italy could be recognized with high geographical resolution, i.e. at local level. Specifically, we analyzed the local spread of the main PFGE clonal group, responsible for more than 60% of human infections in the study area, that did not show any geographical differentiation by PFGE within Northern Italy, i.e. the macro-area considered in the study. Both discrete and continuous phylogeography highlighted the existence of two main geographically-restricted clades: a Southern clade corresponding to the Po Valley and a Northern clade corresponding to the Pre-Alps area. Furthermore, the phylogeographical analyses suggested that the most probable site of origin of the clone was in an area of the Po Valley at the confluence of the Po and Ticino rivers, one of the most important Italian wetlands. These findings provide further support to the hypothesis that environmental transmission may play an important role in the ecology of serovar Napoli.

## Introduction

Salmonellosis, caused by *Salmonella enterica*, represents one of the major foodborne diseases in developed and developing countries [1,2]. *Salmonella enterica* is subdivided into 6 subspecies and includes more than 2600 serovars which cause clinical syndromes ranging from asymptomatic carriage to invasive systemic disease. Most serovars associated with disease in humans and other warm-blooded animals belong to subspecies I (*Salmonella enterica* subspecies *enterica*) and can be divided in two main groups, typhoidal and non-typhoidal serovars. Typhoidal serovars (Typhi and Paratyphi A, B, C) are responsible for typhoid and paratyphoid fevers, while non-typhoidal serotypes typically cause self-limited gastroenteric disease [3,4,5]. Typhoidal *Salmonella*, and particularly *S*. Typhi, represent the prototypical invasive serovars. However, also other serovars are prone to invasiveness, correlating with the presence of two pathogenicity islands–SPI-18 and cdtB islet–in their genome [6,7]. *Salmonella enterica* serovar Napoli (serovar Napoli), an emerging serovar in Europe, has been observed to carry SPI-18 and cdtB islet and to be phylogenetically very close to typhoidal serovars [8].

In Europe, infections caused by serovar Napoli have notably increased over the last years, mainly affecting France, Switzerland, and Italy. During the period 2000–2013, the incidence of serovar Napoli has increased by 256%, becoming the 13^th^ most frequently isolated serovar from humans [9–11]. As regards the Italian situation, a survey performed in Lombardy (Northern Italy) in 2015 showed that serovar Napoli was the third most detected serovar in the region after *S*. Typhimurium and *Salmonella* 1,4,[5],12:i:- [8]. Serovar Napoli caused important outbreaks in the past related to consumption of Italian products including chocolate bars [12] and equine salami [13] among others. More recently, a multi-school outbreak characterized by high rates of hospitalization (30%) and bacteraemia (13%) occurred in Milan in 2014 [14]. Moreover, surveillance data and epidemiological investigations showed that, as for typhoid serotypes, the incubation period of serovar Napoli is typically 7–14 days long (as opposed to 1–4 days of common non-typhoidal seovars) and up to 5.5% of infected patients can develop bacteraemia [8,15]. These new findings underline the virulence potential of this emerging serovar.

In addition to its pathogenic potential, serovar Napoli remains elusive with regard to critical aspects of its epidemiology. Unlike other serovars it appears to be geographically restricted to Northern Italy, Switzerland and Western France, and no specific animal reservoir has been identified so far. A previous case-control study reported that exposure to surface waters of Northern Italian lakes, such as swimming and other recreational activities, increased the risk of infection [16]. In Italy, serovar Napoli has been identified in fresh vegetables produced in the same region [17] in addition to being isolated from wild animals, such as in lizards [18], wild boars [19], river nightingale [20], and foxes [21]. Eight alerts on exported Italian vegetables contaminated with serovar Napoli have been reported in the Rapid Alert System for Food and Feed (RASFF) of the European Commission since 2004 (https://webgate.ec.europa.eu/rasff-window/portal/). This body of evidence is suggestive of a geographically restricted environmental habitat for this serovar, possibly associated with surface water. Recently, through pulsed-field gel electrophoresis (PFGE) analyses, Sabbatucci and coauthors [22] showed a high level of genetic clustering of Italian isolates of serovar Napoli according to their geographic origin with 68% of isolates from Northern Italy belonging to a single PFGE cluster. Based on these findings, we investigated the phylogeographic structure of serovar Napoli in Northern Italy to verify whether a spatio-temporal evolution pattern could be recognized also at local level. The presence of a marked phylogeographical signal at small geographical scale would be indicative of local persistence due to, for instance, animal reservoirs or environmental adaptation. Specifically, we focused on the spatio-temporal diffusion of a successful clonal group of serovar Napoli (defined on the basis of PFGE typing), which is responsible for more than 60% of the human infections by serovar Napoli in the study area. To this purpose, we took advantage of the availability of novel high throughput techniques and genome-wide single-nucleotide polymorphism (SNP) analyses which allow the application of phylodynamic analysis even to slow evolving microorganisms, such as bacteria [23]. In particular, the phylogenetic analysis based on genome-wide SNP has been used to estimate the origin, the distribution, and the mode and time of evolution of different *Salmonella* serotypes [24–26], specific pathovariants within single serotypes [27], and specific multi-drug resistant strains [28,29] on continental or global scales. Phylogeographic analyses have already been performed to investigate the local dissemination and evolution of typhoidal *Salmonella* serotypes within specific geographic regions [30,31], as well as for other enterobacterial pathogens, such as *Shigella sonnei* [32]. In the majority of previous phylogeographic studies a discrete diffusion model has been employed in a Bayesian framework to incorporate geographical information into the phylogeny, grouping the isolates on the basis of the sampling location. Nevertheless, this approach is limited by possible sampling biases due to the fact that the most recent common ancestor (MRCA) origin is drawn from the locations represented in the set. The diffusion of an epidemic occurs in a continuous space, for which reason models to infer diffusion in a bidimensional space, defined by the geographical coordinates, have been recently developed. In this study, we applied both discrete and continuous phylogeographycal models to reconstruct in great detail the dispersion pattern of serovar Napoli in Northern Italy, since its origins.

## Materials and methods

### Description of the study area

**Fig 1:**
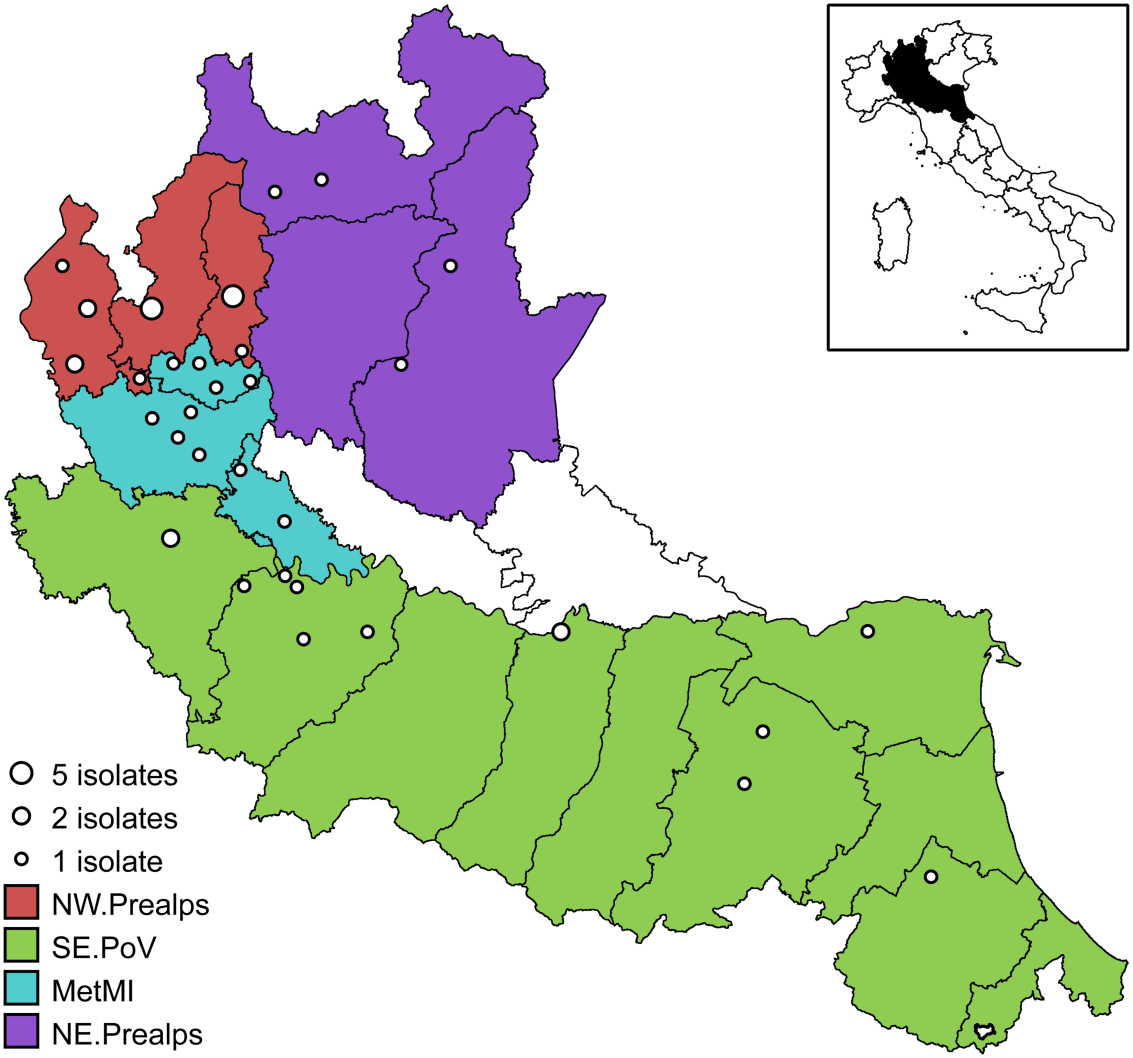
Map of the study area. Open dots represent the locations of the 44 isolates used in the phylogeographical analyses. Colors represent the four areas used in the discrete phylogeography: North Western Prealps (NW.Prealps, red), South-Eastern Po Valley (SE.PoV, green), Metropolitan area of Milan (MetMI, cyan), and North-Eastern Prealps (NE.Prealps, purple). The map of Italy (inset) shows the location of the study area (in black) in the northern part of the country. Map elaborated using data from www.istat.it.

The study area has a surface of 46,317 km^2^ corresponding to the territory of the administrative Regions of Lombardy and Emilia-Romagna in Northern Italy, covering about 15% of the Italian territory. The area is crossed by the Po River and its tributaries, which run from the Alps in the north and from the Apennines in the south (Fig 1). The resident human population is estimated in about 15 million, representing around 24% of the Italian population (data from the Italian National Institute of Statistics).

Data on serovar Napoli isolates from human cases were obtained from the regional laboratory-based surveillance systems integrated in the Enter-Net Italia network. Each case was combined with information on the date of isolation and the municipality of residence (LAU level 2). The residence of each salmonellosis case was geocoded to the municipality centroid, identified by latitude and longitude coordinates.

### Data set and PFGE typing

One hundred and twenty clinical isolates from Lombardy and Emilia-Romagna Regions, 2012–2014, were genotyped by PFGE, according to the PulseNet protocol (https://www.cdc.gov/pulsenet/pathogens/protocols.html). An *XbaI* restriction was performed on genomic DNA before electrophoresis in a Chef Mapper XA system (Bio-Rad, CA, USA). After electrophoresis, we analyzed PFGE patterns using the Bionumerics Software version 7.5 (Applied-Maths, Sint-Martens-Latem, Belgium) and clustered them by the Unweighted-Pair Group Method with arithmetic mean (UPGMA) using Dice coefficient (optimization, 1%; band matching tolerance, 1%). We assumed a coefficient of similarity of more than 0.80 from PFGE types as indicative of a clonal relationship (as in [22]). Then, we developed logistic regression models to evaluate whether isolates coming from different geographic areas belong to different PFGE clusters or sub-clusters, supporting the existence of spatial segregation in serovar Napoli distribution. The analyses were performed through log-likelihood ratio tests (LRT) against the null models.

### Whole-genome sequencing

A total of 43 randomly selected isolates belonging to the main clonal group of serovar Napoli defined on the basis of PFGE typing (representing the 60% of the isolates in the group), and one additional isolate outside the main PFGE cluster but genotypically close to it (more than 70% similarity) were subjected to whole-genome sequencing (WGS), see S1 Table. The random selection of the isolates for WGS was performed through a spatially-stratified sampling design, ensuring a sampling effort of at least 40% of the isolates in each of four areas used in the discrete phylogeography (see Fig 1). Cases with known travels in the five days before the onset of symptoms were discarded during the sampling procedure to avoid recruiting isolates from cases with infection contracted outside the area of the residence municipality. Similarly, cases with *Salmonella* isolation occurred outside the area including the municipality of residence were discarded. The spatial distribution of the 44 sequenced isolates is shown in Fig 1.

Genomic DNA was extracted from overnight cultures using the DNeasy blood and tissue kit (Qiagen, Milan, Italy), spectrophotometrically quantified and controlled for quality. Purified DNA was then processed with the Nextera XT sample preparation kit (Illumina, Inc., San Diego—California, USA) and genomic libraries were sequenced on the Illumina MiSeq platform (Illumina, Inc.) with 2x250 base pairs paired-end runs. Finally, we evaluated the obtained reads for sequence quality and read-pair length using FastQC ver. 0.10.1 [33].

### Bayesian phylogeographic analyses

A core SNPs matrix was generated starting from reads by a reference-free method, namely kSNP3 3.0, with a k-mer length of 21 [34]. Core SNPs were all non-homoplastic SNPs in positions shared by all genomes under analysis. Specifically, kSNP3 removes homoplastic SNPs located in mobile/recombinant regions of the genome since recombinant SNPs can lead to a loss of phylogenetic signals and to errors in the topology of the phylogenetic tree [34]. The derived core SNPs have been used to perform all the phylogenetic analyses.

We estimated the best-fit models, tree topology, evolutionary rates, and phylogeography by using a Bayesian Markov chain Monte Carlo (MCMC) method with the software package BEAST v 1.8.4 [35].

Statistical support for specific clades was determined on the basis of posterior probability (spp) of each monophyletic clade. Four simple parametric models (constant, exponential, expansion and logistic population growth) and the Bayesian skyline plot (BSP) were compared as coalescent models under both a strict and a relaxed (uncorrelated log-normal) clock [35]. MCMC chains were run until convergence (50 million generations, sampling every 5000), which was assessed on the basis of the effective sampling size (ESS ≥ 200) after a 10% burn-in by using Tracer software version 1.6 (http://tree.bio.ed.ac.uk/software/tracer/).

Credibility intervals of the estimates were indicated by 95% highest posterior density (95% HPD). Bayes factor (BF) test was used for best fitting models selection [36]. Only values of 2lnBF ≥ 6 were considered significant [37].

The trees were summarised in a maximum clade credibility, MCC, target tree using the Tree Annotator program after a 10% burn-in. The time of the most recent common ancestor (tMRCA) estimates were calculated as the years before the most recent sampling dates, corresponding to 2011 in this study. The doubling time of the pathogen population was given by the relation *l* = ln(2)/*r* [38] where *r* (representing the population growth rate) was estimated under a simple exponential growth coalescent model by BEAST.

#### Discrete phylogeography

Phylogeographical reconstruction was obtained by the continuous-time Markov Chain process over discrete sampling locations implemented in BEAST [39]. A Bayesian Stochastic Search Variable Selection (BSSVS) model was implemented to identify the most parsimonious description of the phylogeographic diffusion process. The significance of the linkages between locations was tested by BF comparing the posterior and prior probabilities that the diffusion rates between locations would be zero: diffusion rates with BF >3 were considered well supported. The 44 *Salmonella* sequences were assigned to 4 discrete geographic groups, on the basis of their sampling location: a group including isolates sampled in the metropolitan area of Milan (MetMI), a group encompassing the North-Western Prealps (NW.Prealps), which includes the catchment areas of the Como and the Maggiore lakes, and a group encompassing the North-Eastern Prealps (NE.Prealps), which includes the catchment areas of the Garda and the Iseo lakes. Finally, a group corresponding to the Southern and Eastern Po Valley from Pavia to Ferrara (SE.PoV) was also considered (Fig 1).

The final tree was visualised using FigTree version 1.4 (http://tree.bio.ed.ac.uk/software). Significant migration rates and visualization were obtained by using SPREAD, (http://www.kuleuven.be/aidslab/phylogeography/SPREAD.html).

#### Continuous phylogeography

Since the sampling localities were known (S1 Table) and georeferenced by coordinates, the diffusion process in continuous space was also investigated, by using the previously described method implemented in BEAST [40]. The coordinates of the internal nodes and of the root of the tree, were estimated under a strict Brownian diffusion model compared to three Relaxed Random Walk (RRW) models, relaxing the diffusion rate constancy assumption [41]. Three RRW models (assuming Gamma (GA), Cauchy (CA) and Lognormal (LN) distribution of the diffusion rates) were compared through Bayes factor, estimating marginal likelihood, by path-sampling (PS) and stepping-stone approaches (SS) [42]. The phylogeny was spatially projected and converted in the keyhole markup language (KML) for visualization of the dispersal over time in Google Earth (http://earth.google.com/). Uncertainty in ancestral location estimation was represented by KML polygons [41].

#### Accession Numbers

Raw reads of the 44 sequenced isolates from the study were deposited at EBI under project no. PRJEB9682.

## Results

### PFGE typing and whole genome sequencing

**Fig 2:**
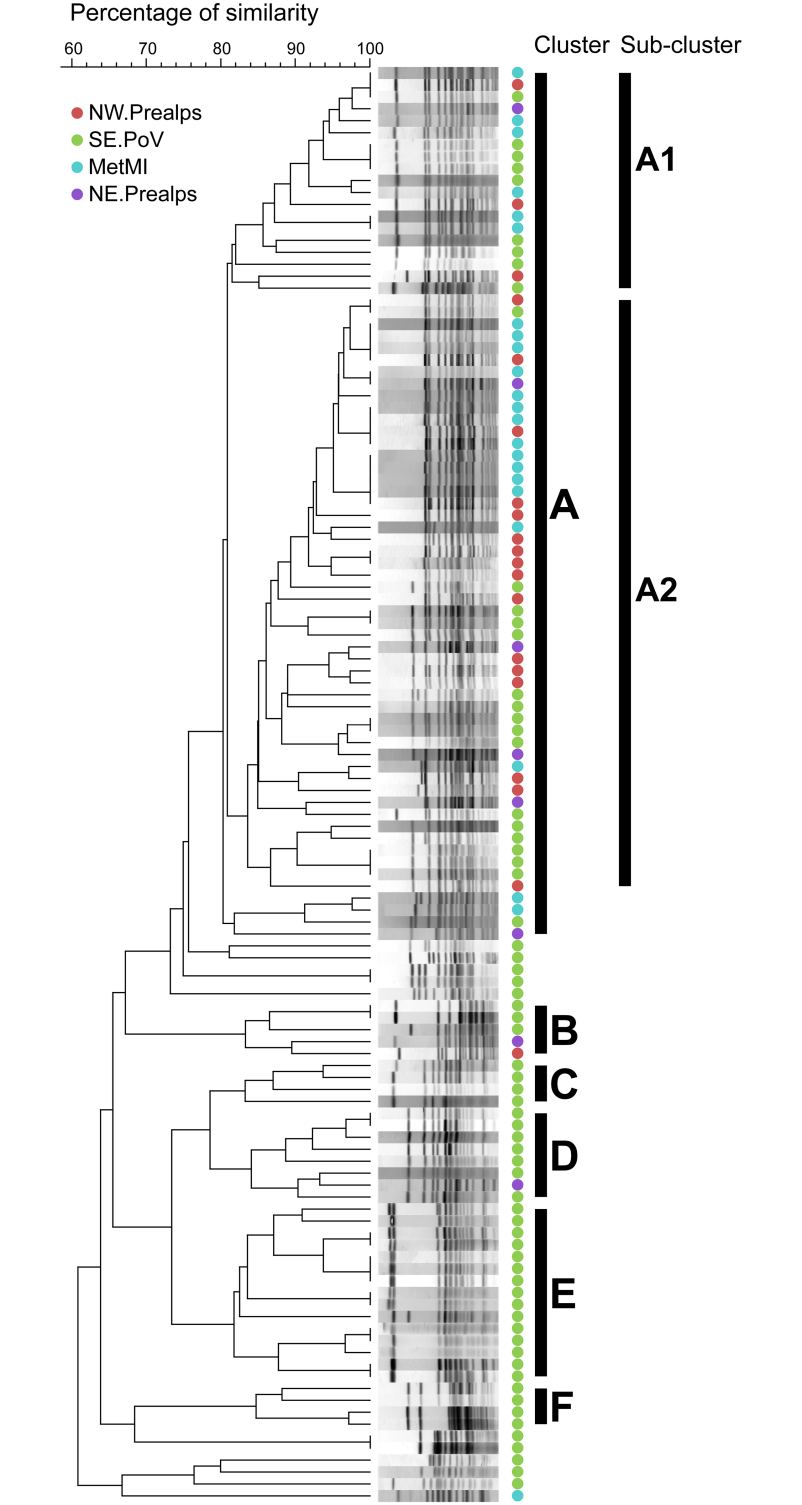
PFGE clustering. PFGE Dendrogram of 120 clinical isolates from Northern Italy. PFGE clusters were identified with letters from A to F. Genetic analysis was based on 80% similarity. Cluster A is subdivided into two main sub-clusters, A1 and A2. The geographic areas of isolation are color-coded: North Western Prealps (NW.Prealps, red), South-Eastern Po Valley (SE.PoV, green), Metropolitan area of Milan (MetMI, cyan), and North-Eastern Prealps (NE.Prealps, purple).

PFGE analysis highlighted the presence of five clusters (Fig 2): one main cluster (A) including 61% of the study isolates and five minor clusters (B to F), including from 3% to 12% of the study isolates. The logistic regression analysis based on PFGE data showed that the geographical areas of origin significantly predicts whether isolates belong to cluster A or not (*n* = 120, LRT, *p* < 10^−8^), with more than 90% of isolates from NW.Prealps, MetMI, and NE.Prealps clustering in A, as shown in Fig 2. Furthermore, Cluster A can be subdivided in two main sub-clusters, A1 and A2, representing about the 95% of isolates in A (and with a percentage of similarity between sub-clusters slightly higher than the 80%). The logistic regression analysis on sub-clusters within A showed that the geographical areas of origin are not significantly associated to the genotypes corresponding to the different sub-clusters (*n* = 69, LRT, *p* = 0.47).

Average sequencing coverage was 97x (2 x 250 bp) (see S1 Table) with >75% of total bases with QC > = 30. Raw reads were checked for quality and processed using KSNP3 to produce a SNP matrix of 2542 SNPs, shared by all 44 sequenced serovar Napoli isolates.

### Bayesian phylogeny and phylogeography

The comparison of the coalescent priors under relaxed and strict molecular clock models showed that the uncorrelated lognormal relaxed clock (2lnBF strict *vs* relaxed clock = 21.76) and the exponential growth model (2lnBF exponential *vs* constant population size = 203.45) were significantly better than all of the alternative models.

A mean evolutionary rate of 1.09 x 10^−3^ subs/site/year with a credibility interval between 4.28 x 10^−5^ and 2.19 x 10^−3^ subs/site/year was estimated for the dataset of 2542 SNPs. Because we used only variable sites, we calculated an evolutionary rate of 5.7 x 10^−7^ [2.2 x 10^−8^–1.14 x 10^−6^] sub/site/year (considering a genome length of 4,888,887 ntds-NCBI Reference Sequence: NC_010102).

The evolutionary population dynamic analysis provided an estimate of the exponential growth rate (*r*) of 0.119 year^-1^ (95% HPD: 0.08–0.25) corresponding to a doubling time of the pathogen population estimated between 3 and 9 years (a mean of 6 years).

#### Discrete phylogeography

**Fig 3:**
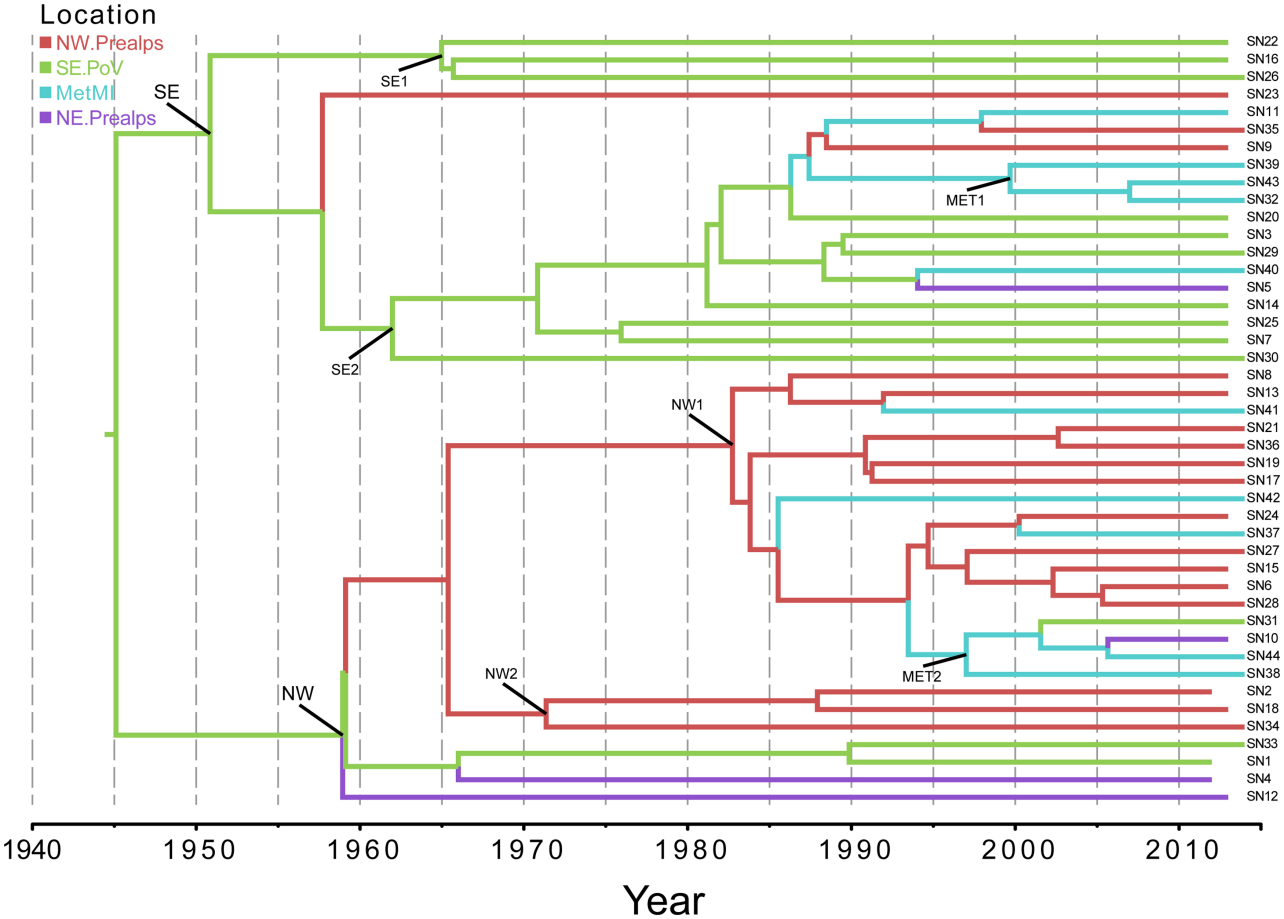
Discrete phylogeography. Phylogeographic analysis of 44 isolates of serovar Napoli in Northern Italy. The branches of the maximum clade credibility (MCC) tree are color-coded on the basis of the most probable location of the descendent nodes: NW.Prealps (red); SE.PoV (green); MetMI (cyan); and NE.Prealps (purple). The scale at the bottom of the tree represents calendar years. The main geographical clades are highlighted (see also Table 1).

Fig 3 shows the phylogeographical MCC tree of the entire dataset. The analysis of the tree confirmed the existence of two highly significant clades: one mainly including the isolates sampled in the South-east Po Valley area (clade SE, spp = 0.92) and the other encompassing the North-Western Prealps isolates (NW, spp = 1). The isolates of the Milan metropolitan area tended to be interspersed in both clades, forming only small subclades of no more than 3 isolates (MET1 and MET2). The most probable location of the tree-root was in South-east Po Valley region. The mean tMRCA of the tree-root was estimated to be 69 YA (95%HPD: 16–165 YA), corresponding to 1945 as a mean date. In Table 1 are reported the tMRCAs and the most probable locations of the main clades and subclades. The South-east Po Valley subclades have tMRCA estimates between 1950 and 1965, while the North-Western Prealps subclades between 1960 and 1980. Two well-supported diffusion rates were identified by BF analysis: between the Metropolitan area of Milan (MetMI) and the South-East Po Valley area (BF = 5621) and between the MetMI the North-Western Prealps area (BF = 7).

**Table 1:** Estimated times of the most recent common ancestors (tMRCAs) of the main clades and 95% credibility intervals (HPD), with calendar years, most probable locations, and state posterior probabilities (spp) derived from discrete phylogeography of the 44 genomes of serovar Napoli.

Node	Clade	Subclade	tMRCA	Upper HPD	Lower HPD	Locality	spp
**ROOT**			1945	165	16	SE.PoV	0.92
	**SE**		1951	151	14	SE.PoV	0.99
		**SE1**	1965	116	10	SE.PoV	1
		**SE2**	1962	126	11	SE.PoV	1
		**MET1**	2000	35	3	MetMI	0.98
	**NW**		1959	133	13	SE.PoV	0.77
		**NW1**	1983	75	8	NW.Prealps	0.99
		**NW2**	1971	102	9	NW.Prealps	0.98
		**MET2**	1997	40	4	MetMI	0.67

#### Continuous phylogeography

**Fig 4:**
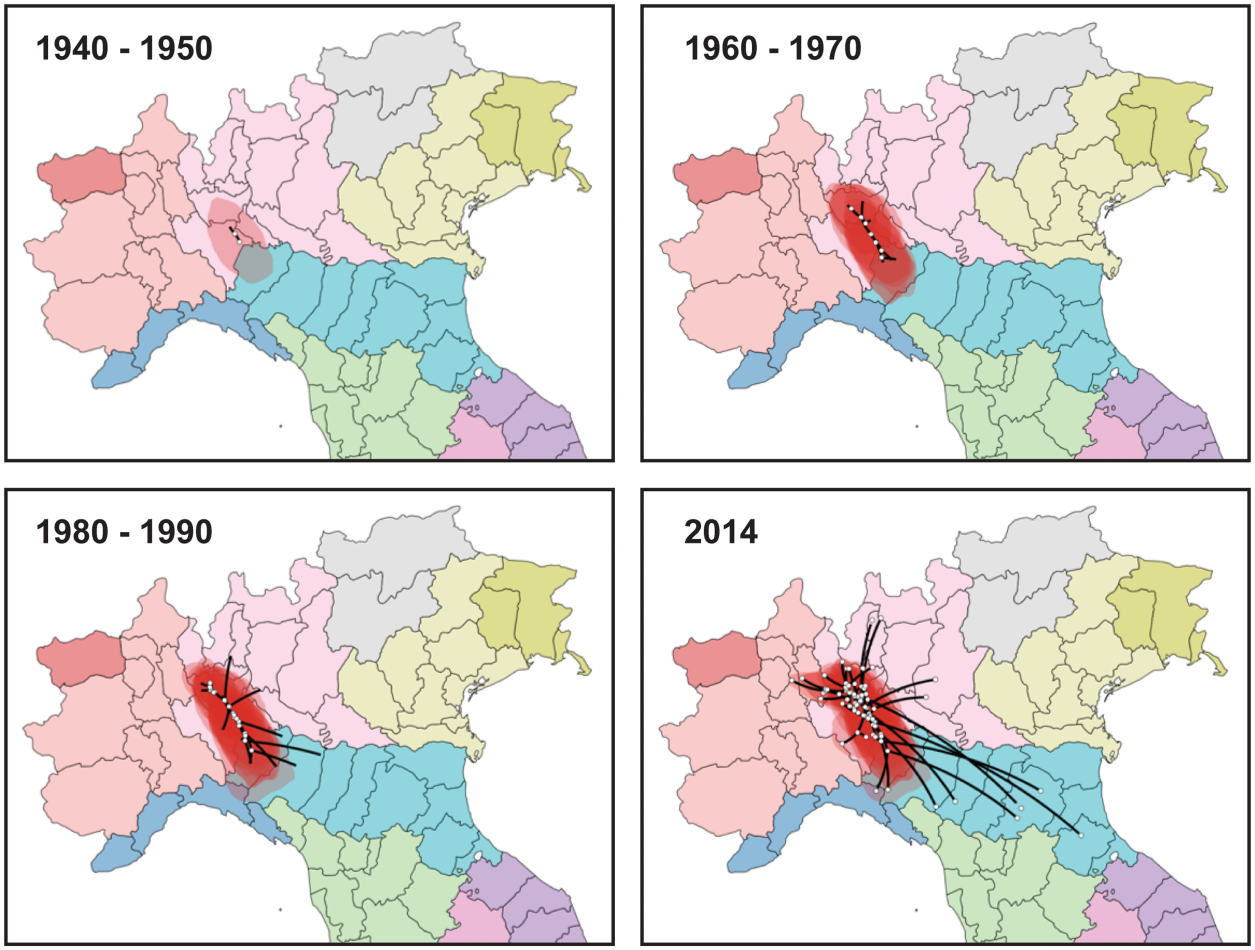
Spatio-temporal dynamics of the Salmonella Napoli in Italy. The inferred spatiotemporal dynamics of Salmonella Napoli in Northern Italy. The figure summarizes the most significant migration links in the interested area. More detailed results are reported in S1 Video.

A phylogeographic analysis in continuous space has been performed using known coordinates of the sampling locations. The comparison of marginal likelihood estimates for the different diffusion models showed always significantly better fit of RWW models than BD model: specifically, RWW-Gamma (2lnBF = 18597.29 by PS and 18597.57 by SS for comparison against BD) and for RWW-lognormal (2lnBF = 18598.43 by PS and 18596.53 by SS always by comparison with BD). The LN-RRW diffusion model was shown to fit better our data and was employed for phylogeographic reconstruction. Fig 4 represents the projection in calendar time scale of the tree branches onto a map of the interested geographic area, georeferenced by the coordinates estimated for the internal nodes and the tips of the tree (See also the animation in S1 Video). The most probable location of the tree root was placed at 45°12’ N and 9°18’ E corresponding to the Southern Lombardy, near the confluence between the Ticino and Po rivers. The infection then spread simultaneously towards the North-West and the South-East. In particular, Northwest diffusion followed the courses of the Ticino and Adda rivers, reaching the metropolitan area of Milan as soon as the early 1950s and expanding to the Northern area until reaching the region of the great lakes of Lombardy in 2000s. The Southeast dispersal apparently followed the Po River, reaching Piacenza in 1960s-1970s. In the late 1980s-1990s it reached the provinces of Parma, Reggio Emilia, Modena of Emilia-Romagna up to Bologna in the 2000s. Most external branches reached the area near the Adriatic Sea (Forlì) only recently (2014). Overall, the spread affected an area of more than 150 km from North to South and 250 from West to East, at an estimated diffusion rate of 2.8 (0.11–5.5) km/year.

## Discussion

We analyzed the time and mode of evolution of the dominant clone of serovar Napoli in Northern Italy considering 2542 core SNPs of 44 isolates and we have estimated a mean evolutionary rate of 5.7x10^-7^ sub/site year^-1^, corresponding to a mean 2.7 substitutions/genome year^-1^. This value is at the upper limit of previous estimates obtained on different *Salmonella enterica* serovars, varying from 0.8 to about 6.1 x10^-7^ sub/site year^-1^ [25,26,29,43,44]. It was previously observed that human-restricted serovars (such as *S*. Typhi and Paratyphi A) have evolutionary rates on average lower (0.8–2 x10^-7^ sub/site year^-1^) than those estimated for less restricted serovars such as Kentuky, Agona, Gallinarum (3.95-6x10^-7^-sub/site year^-1^) [25,26,29,43,44].

Moreover, it is well known that the time interval over which the bacterial sequences have been sampled affects the estimates of evolutionary rate [45]. In particular, short sampling intervals such as those of individual outbreaks, tend to yield high evolutionary rates due to the accumulation of persisting slightly deleterious mutations, which would be removed by purifying selection in longer time intervals. On the other hand, the use of evolutionary rates estimated on a larger time-scale tend to under-estimate the tMRCAs, when applied to an epidemiological scale [23].

In this study we focused on the local spread of a dominant serovar Napoli clone, restricted to a relatively homogenous area and over a small period of time (between 2012–2014). However, given that we reconstructed events occurred in relatively recent times (the root of the tree went back to the mid-1900s), and thanks to the availability of epidemiological data supporting the phylodynamic reconstruction, we can be confident in our results.

On the basis of our phylogeographical analysis, serovar Napoli strains causing recent infections in Northern Italy shared a common ancestor originating about a mean of 69 years ago most probably in an area located in the South-East Po Valley.

The discrete phylogeography confirmed the existence of two highly significant clades defined on a geographical base: a Southern clade corresponding to the Po Valley and a Northern group corresponding to the Pre-Alps area. As expected, given the origin of the serovar Napoli diffusion in the Po Valley, the Southern clade was older (1950–65) than the Pre-Alps one (1960–1980). A third geographical group was identified in the metropolitan area of Milan, but strains isolated in that area formed different subclades, being included in both the Southeastern and the Northwestern clades (see MET1 and MET2 in Fig 3), dating to more recent times (mean tMRCA estimates 1986 and 1997, respectively). The analysis of the discrete diffusion rates showed the central role played by the Milan surrounding metropolitan area, which was significantly linked to all the other localities, suggesting the occurrence of serovar Napoli spread between the Milan area and the other localities.

Interestingly, surveillance and literature data show that the outbreak in England and Wales, associated with the consumption of chocolate bars produced in the North-Western hinterland of Milan [12], occurred in 1982. That time was immediately after the period during which the North-West clade originated according to the results of the phylogeographical analysis. More recently, in 2014, a multi-school outbreak occurred just 20 km away from the location where the chocolate bars were produced [14]. The infection vehicle of this outbreak was not identified, but its causative strain belongs to the North-Western clade. Unfortunately, the 1982 outbreak strain is no longer available to check if it was part of the same clade.

The main limitation of discrete models for incorporating geographical information into the phylogeny is the possible sampling bias due to the fact that the MRCA origin is drawn from the locations represented in the set. In reality, the spread of an infectious agent occurs in a continuous space and is best represented by models inferring diffusion in bidimensional space, defined by the geographical coordinates.

The results of the phylogeographical analysis in continuous space suggested, in agreement with the discrete analysis, that the most probable origin of the diffusion of the considered clone of serovar Napoli was in an area of the Po Valley at the confluence of the Italian greatest rivers, Po and Ticino, one of the most important Italian wetlands. From there, serovar Napoli dispersed into two different directions. The first followed the course of the Po River, progressing towards the Southeast and reaching all the different urban centers of Emilia-Romagna starting from Piacenza (extreme North-West of the region) in the years 1960–70, down to Bologna in 2000s. Only very recently (2014) the strain reached the Italian Adriatic coast at the extreme South-East of the region. Along the second path, the spread reached the metropolitan area of Milan as early as the 1950s, shortly after the origin, then followed the course of other major Italian rivers, such as Ticino and Adda, reaching the region of the great Northern Italian lakes in the 2000s. These spatial migration paths showed the central role played by the surrounding area of Milan for the diffusion of the pathogen both to the north-west and to the south-east, in agreement with the results of the discrete analysis.

Notably, the area from where serovar Napoli began to spread corresponds to the confluence of the Ticino river into the Po river. Moreover, nearby the confluence with Po river, Ticino receives a tributary, the Olona, which precisely crosses the area affected by the two already mentioned outbreaks of 1982 and 2014. The diffusion of the North-West clade into the Prealpine area corresponds to the time of the infection alert coming from the Canton of Ticino (2002), an area of Switzerland that borders Italy and is crossed by the Ticino river which, at the border between the two countries, forms Lake Maggiore, of which Ticino forms the tributary and then the emissary [9].

Overall, the phylogeographical analysis suggests the catchment area of the Ticino river as the area where the diffusion of serovar Napoli in Northern Italy started, and supports the hypothesis that surface waters may play an important role in the diffusion of serovar Napoli. Oggioni and colleagues conducted a case-control study in Lombardy and documented an odds ratio of 3.82 (95% CI = 1.03–14.19) for exposure to surface waters during play, recreation and sporting activities. Conversely, they did not detect any association with food vehicles [16]. Furthermore, in a primary school in another northern Italian region, an outbreak related to the consumption of contaminated water occurred in 2011 [46].

Notably, a recent PFGE-based study on serovar Napoli in Italy [22] identified different clusters of isolates matching with three longitudinally defined macro-areas (specifically, Northern, Central, and Southern Italy). Interestingly, the clusters of that study included isolates of diverse origin from the same areas, namely human, animal, and environmental origin. We too found that PFGE could identify some spatial structure in serovar Napoli distribution (see clusters B-F in Fig 2 restricted mainly to the SE.PoV area). However, in our study, PFGE was not able to detect a spatial structure inside the main cluster of serovar Napoli in Northern Italy (sub-clusters A1 and A2 in Fig 2) while the more advanced phylodynamic techniques used provided a clear picture of the spatial and temporal evolution inside this clone at local level.

All the above observations, including the phylogeographical analysis, support the hypothesis of an important role of surface waters in the transmission of serovar Napoli infection following the pattern “wild animals-waters-(food)-man (as already suggested in [11], [16], and [22]). Consistent with this scheme are the reported isolations from wild animals in Italy such as lizards [18], wild boars [19], river nightingale [20], and foxes [21]. The mentioned species are strictly territorial animals. This may explain the small-scale diffusion pattern of genetically similar strains of serovar Napoli in the study area.This serovar could be adapted to one or more wild animal species able to contaminate surface waters and consequently lead to human infections by direct contact or by contamination of irrigated crops and food chains. Consistent with this scenario, in 2014 a RASFF (Rapid Alert System for Food and Feed) notification reported the presence of serovar Napoli in Italian rucola salad (RASFF 2014.1410). Considering that surface waters probably do not favor microbial replication and the reaching of high microbial loads, it could be hypothesized that infection by serovar Napoli is possible following the ingestion of low bacterial loads. This hypothesis agrees with the low infective dose reported for the aforementioned outbreak occurred in the United Kingdom in 1982 (10^2^ CFU per g of chocolate bars) [47], and represents an important pathogenic similarity with typhoid serovars. Such serovars have also been recently shown by Huedo and colleagues to be highly genetically related to the 2014 multi-school outbreak strain of serovar Napoli included in this study [14].

In agreement with other studies, our analysis highlighted the existence of spatially restricted clusters suggesting the adaptation to different environments and/or ecological niches. Accordingly, Fisher and colleagues showed that isolates from different sources correlated with each other according to the isolation area [9], while Graziani and colleagues observed the local circulation of different clones, which were able to persist in the same environment for many years [11]. The existence of a spatial structure at local scale is a necessary condition to support the hypothesis of wildlife reservoirs for serovar Napoli infections in human. To test this hypothesis further investigations are required on whether local transmission of serovar Napoli is maintained within wildlife and human populations separately or if frequent host species jumps occurred. The presence of frequent host species jumps would demonstrate the presence of common sources of infection, confirming the environmental/wildlife origin of a significant number of human infections by serovar Napoli. Phylodynamics techniques, like those we used, represent ideal approaches to address this issue [48,49].

In the study, we used a limited number of isolates originated from a passive surveillance system which may be susceptible to spatial/temporal reporting bias, suggesting caution in the interpretation of the results. While reporting bias is known to negatively affect the reliability of space-dependent estimates of classical epidemiological parameters (such as incidence, mortality, etc.), its role seems less critical in affecting the accuracy of spatial structure determination of genomic clusters, as we did in this work.

With regard to the spatial attribution of infections, small-scale movements of the cases included in the analysis may play a critical role. Generally, this detailed information is not collected by surveillance systems, as was the case in our surveillance. To mitigate the possibly negative effect of spatial mis-attributions, we excluded from the phylogeographical analysis cases with known travel history and those not having isolation and residence within the same area. In addition, we claim that if a spatial structure of the infection does exist, random mis-attribution of infection locations could lead to leaving the structure undetected (i.e. type II errors). Conversely, it is less likely that random mis-attributions can lead to identifying an unexisting spatial structure (i.e. type I errors). On the other hand, we have no indication of systematic mis-attributions due to the organization of the health system. Specifically, all the four areas had their own medical facilities for the diagnosis and treatment of Salmonellosis infections, suggesting that no systematic movement of cases occurred across the areas. Consequently, considering that our study identified a clear spatial patter of serovar Napoli infections, we are confident that the discussed caveat on potential spatial mis-attributions did not invalidate our conclusions.

This study demonstrates the importance of combining whole genome sequencing with the most advanced population-based phylogenomic analyses in tracing the origin and pathways of slow-evolving environmentally- and directly-transmitted pathogens to better understand their epidemiology.

## Supporting information

S1 Table Sequences included in the study. Serovar Napoli isolates sequenced in this study with related epidemiological information and WGS coverage.
(XLSX)

S1 Video Continuous phylogeography visualization. Animated visualization of the continuous pattern of Serovar Napoli dispersion in Northern Italy in the years 1950–2014.
(KML)
